# MAP17 contributes to the tumorigenesis of papillary thyroid carcinoma by activating the AKT signaling pathway

**DOI:** 10.20945/2359-4292-2024-0342

**Published:** 2025-02-28

**Authors:** Zhen-Hua Tian, Rui Huang, Gang-Qiang Li, Yong-Xue Zhu

**Affiliations:** 1 Department of Head & Neck Surgery, Fudan University Shanghai Cancer Center, Shanghai China; 2 Department of Oncology, Shanghai Medical College, Fudan University, Shanghai, China; 3 Department of Pathology, Naval Medical Center, Naval Medical University PLA, Shanghai, China; 4 Department of General Surgery, Naval Medical Center, Naval Medical University PLA, Shanghai, China

**Keywords:** Papillary thyroid carcinoma, MAP17, AKT

## Abstract

**Objective:**

This study investigates the role of membrane-associated protein 17 (MAP17) and the Akt signaling pathway in the progression of papillary thyroid carcinoma (PTC).

**Materials and methods:**

We conducted a series of in vitro experiments using PTC cell lines (HTori-3 and TPC-1). Cells were divided into three groups: control, MAP17 inhibitor negative control (NC), and MAP17 inhibitor treatment. Cell viability was assessed at 0, 24, 48, and 72 hours using the Cell Counting Kit-8 (CCK-8) assay. Apoptosis levels were measured by flow cytometry, and protein and mRNA expression of MAP17, phosphorylated Akt (p-AKT), and Akt were analyzed by Western blot and qRT-PCR.

**Results:**

Cell viability in the control, MAP17 inhibitor NC, and MAP17 inhibitor groups increased significantly over time (P < 0.05). Notably, in both HTori-3 and TPC-1 cells, the MAP17 inhibitor significantly reduced cell viability compared to the control and NC groups at 24, 48, and 72 hours (P < 0.05). Furthermore, apoptosis levels were significantly higher in the MAP17 inhibitor group compared to the control and NC groups (P < 0.05). Western blot and qRT-PCR analyses revealed that MAP17 and p-Akt protein and mRNA levels were significantly higher in the control and NC groups compared to the MAP17 inhibitor group (P < 0.05). However, no significant differences in total Akt protein or mRNA levels were observed across groups.

**Conclusion:**

Our findings suggest that MAP17 and the Akt signaling pathway play a crucial role in promoting the progression of PTC. Inhibition of MAP17 suppresses cell viability and induces apoptosis, indicating that MAP17 may be a promising therapeutic target for PTC. The data also highlight the potential for targeting the MAP17-Akt axis in developing future treatments for PTC.

## INTRODUCTION

Papillary thyroid carcinoma (PTC) represents the most prevalent form of thyroid cancer, making up about 80% of all cases (^1^). Originating from the follicular cells of the thyroid gland, PTC is marked by its slow progression and generally positive prognosis when compared to other thyroid cancers (^2^). This type of cancer is more commonly found in women than in men, with a higher incidence among individuals aged between 30 and 50 years old (^3^). Symptoms of PTC can include a noticeable thyroid nodule, hoarseness, neck discomfort, or swollen lymph nodes. Known risk factors include exposure to ionizing radiation, particularly during childhood or due to nuclear accidents. Diagnosing PTC involves a combination of imaging techniques, fine needle aspiration biopsy of the thyroid nodule, and histological analysis following thyroidectomy (^4^). The main treatment approach is surgical, often entailing a total or near-total thyroidectomy (^5^). Depending on the disease’s severity, radioactive iodine therapy may be advised to eliminate any residual thyroid tissue or cancer cells. Post-surgery, thyroid hormone replacement therapy is typically necessary. Despite advancements in treatment and prevention, a complete cure for PTC remains elusive, highlighting the importance of continued research to unravel the complex mechanisms underlying PTC (^6^). Interestingly, there are links between MAP17 and the AKT signaling pathway and the development of PTC, though their exact roles in the disease’s pathology are not fully understood. In our research, we have highlighted the potential association between MAP17 and the AKT signaling pathway in the development of papillary thyroid carcinoma (PTC). Papillary thyroid carcinoma is the most common type of thyroid cancer, accounting for approximately 80% of all thyroid cancer cases. Although PTC generally has a good prognosis, its pathogenesis remains not entirely clear, especially at the molecular level. In recent years, studies have found that MAP17 may play an important role in the occurrence and development of PTC, particularly through the regulation of the AKT signaling pathway. The AKT signaling pathway plays a crucial role in cell proliferation, survival, and metabolism, and its abnormal activation is closely related to the occurrence of various cancers. Although existing studies suggest a link between MAP17 and the AKT signaling pathway, the specific mechanisms have not been fully elucidated. Therefore, an in-depth exploration of the roles of MAP17 and the AKT signaling pathway in PTC will not only help understand the biological characteristics of PTC but also provide potential targets for the development of new therapeutic strategies. This study aims to fill this knowledge gap by experimentally investigating the interaction between MAP17 and the AKT signaling pathway, revealing their specific mechanisms in the development of PTC. This will provide new insights for future clinical treatments and promote progress in thyroid cancer research and therapy. By focusing on MAP17 and the AKT signaling pathway, we hope to provide deeper insights into the study of papillary thyroid carcinoma and promote scientific development in the related field.

MAP17, also known as PDZK1IP1 (PDZ domain containing 1 interacting protein), encodes a protein that plays a role in a variety of cellular functions, such as cell growth, movement, and viability (^7^). MAP17 has been implicated in the progression and metastasis of several malignancies, including thyroid cancer. In papillary thyroid carcinoma (PTC), studies have shown that MAP17 expression is upregulated in PTC tissues compared to normal thyroid tissue (^8^). Elevated levels of MAP17 are associated with more aggressive tumor behavior, increased risk of lymph node metastasis, and poorer prognosis in PTC patients. The exact mechanisms by which MAP17 promotes tumor progression in PTC are still being elucidated (^9^). However, it is hypothesized that MAP17 may augment cell proliferation, invasion, and resistance to programmed cell death via diverse signaling pathways. Further investigation is needed for a comprehensive understanding of the role of MAP17 in PTC and its potential as a therapeutic target or prognostic marker. Investigating the molecular mechanisms of MAP17 in PTC may lead to novel treatment strategies for patients with aggressive or recurrent PTC.

The PI3K-AKT pathway, also known as the AKT signaling pathway, plays a crucial role in controlling various cellular processes such as cell growth, proliferation, and survival, metabolism, and migration. Dysregulation of the AKT pathway is commonly associated with cancer development and progression, including thyroid cancer (^10^,^11^). Activated AKT in PTC promotes cell proliferation by stimulating the expression of genes involved in cell cycle progression and inhibiting apoptosis (^12^). AKT regulates key downstream effectors which play a role in protein synthesis and cell growth (^13^). dysregulated AKT signaling pathway in PTC contributes to tumor progression and aggressiveness, highlighting the importance of understanding and targeting this pathway for the development of novel therapeutic approaches in the management of PTC.

The PI3K-AKT signaling pathway, a critical regulator of various cellular processes, plays an essential role in cell growth, proliferation, survival, metabolism, and migration. This pathway is frequently dysregulated in a variety of cancers, including thyroid cancer, contributing to tumor initiation and progression. In particular, activated AKT has been shown to promote cell proliferation by stimulating the expression of genes involved in cell cycle progression while inhibiting apoptosis. AKT signaling also regulates several downstream effectors that are crucial for protein synthesis, cell survival, and growth, making it a central hub for cellular regulation. In the context of papillary thyroid carcinoma (PTC), the most common form of thyroid cancer, dysregulated AKT signaling is implicated in tumor progression and aggressiveness. Recent studies have highlighted the critical role of AKT in mediating the proliferative and anti-apoptotic effects that drive PTC progression. For example, activated AKT in PTC cells promotes tumor growth by facilitating the expression of genes involved in cell cycle regulation and by suppressing programmed cell death. Dysregulation of the PI3K-AKT pathway has also been associated with increased metastatic potential and resistance to therapy in various cancers, including PTC. This underscores the importance of understanding the molecular mechanisms underlying AKT signaling in cancer to identify potential therapeutic targets for intervention. Recent literature further supports the significance of the PI3K-AKT pathway in cancer biology. For instance, a study (^14^) demonstrated that targeting the EGFR-mediated PI3K-AKT pathway can inhibit lung metastasis in triple-negative breast cancer (TNBC), emphasizing the role of AKT signaling in metastasis regulation. Similarly, a study (^15^) highlighted how targeting the PI3K-mTOR pathway, in conjunction with MMP2/9, can prevent migration in TNBC, illustrating the relevance of the PI3K-AKT signaling axis in controlling tumor migration and invasion. These findings further reinforce the critical role of AKT signaling in cancer metastasis and progression, suggesting that therapeutic strategies aimed at modulating this pathway could provide new avenues for treating aggressive cancer types, including PTC. Despite the well-established role of AKT signaling in cancer, the precise molecular mechanisms by which it contributes to PTC development and progression remain incompletely understood. Therefore, the aim of this study is to investigate the functions of MAP17, a potential regulator of AKT signaling, in the context of PTC. Understanding the interaction between MAP17 and AKT could provide valuable insights into the molecular drivers of PTC and pave the way for the development of targeted therapies for this common and clinically challenging cancer. In summary, targeting the PI3K-AKT signaling pathway offers a promising strategy for therapeutic intervention in PTC and other malignancies. This study seeks to elucidate the role of MAP17 in regulating AKT signaling and its impact on the progression of PTC, with the goal of identifying potential new targets for treatment.

Prior studies have identified MAP17 a gene that encodes a protein involved in PTC process, and dysregulation of the AKT pathway is commonly associated with PTC development and progression (^8^). Nevertheless, the intricate mechanisms by which MAP17 modulates the AKT pathway in PTC remain to be elucidated, necessitating further investigation to pinpoint the exact processes. Here, we conducted an in vitro study to investigate the role of MAP17, and AKT signaling pathway in tumorigenesis of PTC.

## MATERIALS AND METHODS

### Cell culture and treatment

The normal human normal thyroid HTori-3 cell and PTC cell lines TPC-1 were obtained from PromoCell Co., Ltd. These cells were maintained in DMEM medium (Thermo Fisher, USA) by supplementing with 1% penicillin/streptomycin and 10% fetal bovine serum from Biologic Industries, the cells were cultured at 37 °C in a 5% CO_2_ humidified atmosphere. The cells were cultured in DMEM supplemented with penicillin/streptomycin and FBS, which are commonly used to support cell growth and provide essential nutrients. To investigate the role of MAP17 in PTC cell lines, we divided the cells into three groups: control, MAP17 inhibitor negative control (NC), and MAP17 inhibitor groups. The control group served as a baseline reference without treatment. In the MAP17 inhibitor NC group, cells were transfected with inhibitor NC of MAP17, while in the MAP17 inhibitor group, cells were transfected with an inhibitor of MAP17. Further, to elucidate the mechanism by which MAP17 regulates AKT signaling pathway, we utilized the same grouping strategy: control, MAP17 inhibitor NC, and MAP17 inhibitor groups. Each group was treated correspondingly.

### CCK8 assay

In the CCK-8 assay, cells are generally planted in a 96-well plate and exposed to various experimental conditions. Following a defined incubation duration, the CCK-8 reagent is introduced to each well and permitted to interact with the cells. This interaction between the CCK-8 reagent and cellular dehydrogenases generates the formazan dye, with its intensity being directly related to the quantity of living cells.

### Flow cytometry assay

Following the manufacturer’s guidelines, the collected cellular specimens were assessed using flow cytometric analysis. The samples were treated with fluorescently-labeled Annexin V and Propidium Iodide (PI) in the absence of light. Subsequently, the apoptotic rates for each group were analyzed using a flow cytometer.

### Western blotting

Proteins from the cells were resolved by 10% SDS-PAGE and subsequently transferred onto a PVDF membrane. To minimize non-specific interactions, the membrane was washed with TBST. Primary antibodies targeting the protein of interest, along with a loading control (β-actin) from (Bioworld Technology, Inc., China) were applied to the membrane and incubated at 4 °C overnight. Post-incubation, the membrane was washed with TBST to eliminate unbound primary antibodies and non-specific interactions. A secondary antibody, provided by (Bioworld Technology, Inc., China) was then incubated with the membrane for 2 hours at ambient temperature. Following this, the membrane was rinsed with TBST to remove any residual secondary antibodies. For protein detection, an ECL luminescent reagent was applied to the membrane, and the resulting bands were subsequently analyzed.

### qRT-PCR

In accordance with the manufacturer’s instructions, total RNA extraction from the cells was carried out using Beyotime TRIzol Reagent (supplied by Shanghai Kanglang Biotechnology Co., Ltd., China). The extracted mRNA was then used to synthesize cDNA with Beyotime’s mRNA Reverse Transcription Kit (Shanghai, China). Quantitative PCR assays were performed using Vazyme Biotech’s SYBR Green PCR Mix (Shanghai, China), and mRNA expression levels were measured on a Real-Time PCR System. The relative expression was calculated through the 2-∆∆Ct method, with β-actin serving as the reference gene. To ensure accuracy, the entire process was replicated three times. The primers for MAP17 were used, Forward: 5’-ATGGAGGAG AGCCGCTCC-3’ and Reverse: 5’-TCAGCAGGCGTGGTCAGG-3’. For AKT: Forward: 5’-AGGGGTACAGCACATTG-3’ and Reverse: 5’-AGTGCCAAGTGCAATCCA -3’. For p-AKT: Forward: 5’-GGCTCCTTTGTTGAC CTGGAT-3’ and Reverse: 5’-AGTTGCGGAAGTGTGAGGGT-3’. Lastly, for β-actin, the sequences were: Forward: 5’-CGGTCAGGTCATCACTATC-3’ and Reverse: 5’-CAGGGCAGTAATCTCCTTC-3’.

### Flow cytometry assay

In adherence to the guidelines provided by the equipment maker, the retrieved cellular specimens were evaluated via flow cytometric analysis. The samples, once collected, were treated with fluorescently-labeled Annexin V and Propidium Iodide (PI) away from light exposure, and their apoptotic rates were subsequently analyzed for each group using a flow cytometry device.

### Statistical analysis

The data analysis was conducted using Prism 8. All measurements were reported as the mean ± standard deviation, with each experiment repeated a minimum of three times. To evaluate the significance of differences between two groups, a t-test was utilized, while comparisons among three or more groups were made using a one-way ANOVA. After conducting ANOVA, it is necessary to perform post hoc tests (such as Tukey or Bonferroni tests) to determine which groups have significant differences. This will help to gain a more comprehensive understanding of the data and reduce the risk of Type I errors (false positives). Additionally, considering the small sample size, it is indeed important to discuss the potential for Type I and Type II errors (false negatives). In cases of small samples, the power of statistical tests may be reduced, so caution should be exercised when interpreting the results. Increasing the sample size or using more conservative statistical methods can be considered to enhance the reliability of the results. A p-value below 0.05 was deemed to indicate statistical significance.

## RESULTS

### Comparison of the cell viability

**Figure 1:**
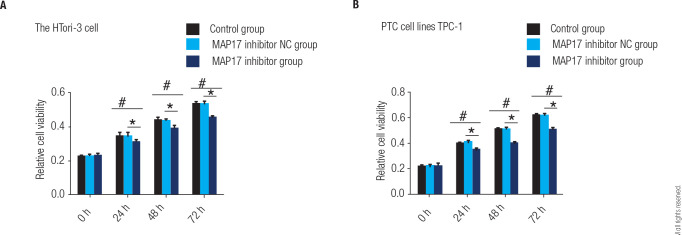
The cell viability for HTori-3 cell line and the PTC cell line TPC-1 were detected using CCK8 assay. Statistical analysis revealed a significant increase in cell viability in the control group compared to the MAP17 inhibitor group (*p < 0.05, #). Additionally, the MAP17 inhibitor NC group showed significantly higher cell viability compared to the MAP17 inhibitor group (*p < 0.05).

The cell viability of MAP17 on proliferation was determined using the CCK8 assay, as shown in **Figure 1**. The HTori-3 cell line and the PTC cell line TPC-1 were divided into control, MAP17 inhibitor NC, and MAP17 inhibitor groups. The CCK8 assay was performed. In HTori-3 cells, the results indicate that cell viability in the control, MAP17 inhibitor NC, and MAP17 inhibitor groups significantly increased with the duration of cell treatment (24, 48, and 72 hours), with a statistically significant P value (P < 0.05). Specifically, in the HTori-3 cells, the cell viability in the MAP17 inhibitor group was significantly lower than that in the control group and the MAP17 inhibitor control group at different treatment times (24 hours, 48 hours, and 72 hours), and this difference was statistically significant (P < 0.05). This result indicates that MAP17 inhibitors have potential application value in suppressing cell viability. (as shown in **Figure 1A**). Similarly, in TPC-1 cells, the results indicate that cell viability in the control, MAP17 inhibitor NC, and MAP17 inhibitor groups significantly increased with the duration of cell treatment (24, 48, and 72 hours), with a statistically significant P value (P < 0.05). The MAP17 inhibitor also inhibited cell viability in this cell line (as shown in **Figure 1B**).

### Comparison of the apoptosis levels

**Figure 2:**
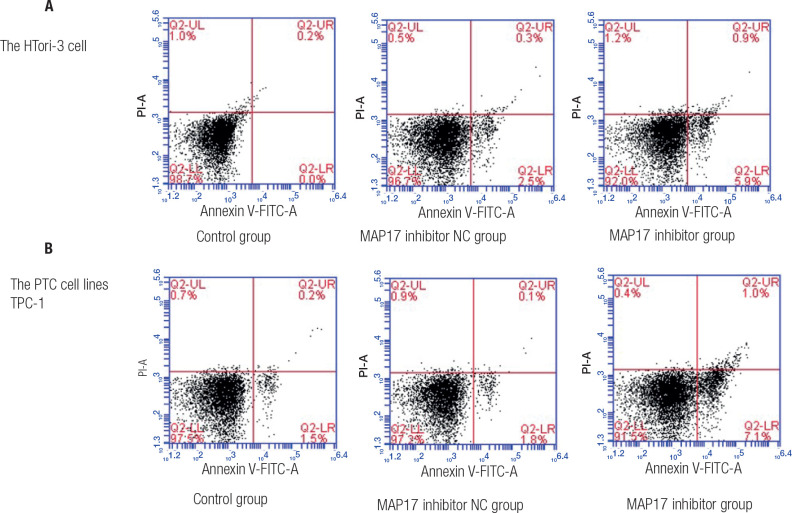
The apoptosis level for HTori-3 cell line and the PTC cell line TPC-1 were detected using Flow cytometry assays.

Flow cytometry assays was utilized to assess the apoptosis level of the HTori-3 cell line and the PTC cell line TPC-1. In the HTori-3 cell, these results suggested that the apoptosis level in control and MAP17 inhibitor NC groups was evidently lower than that in MAP17 inhibitor group (**Figure 2A**), with a statistically significant P value (P < 0.05). In TPC-1, the results suggested that the apoptosis level in control and MAP17 inhibitor NC groups was evidently lower than that in MAP17 inhibitor group (**Figure 2B**), with a statistically significant P value (P < 0.05). These findings demonstrated that inhibitor of MAP17 can increase the apoptosis level.

### Effect of MAP17, AKT and p-AKT protein in PTC

**Figure 3:**
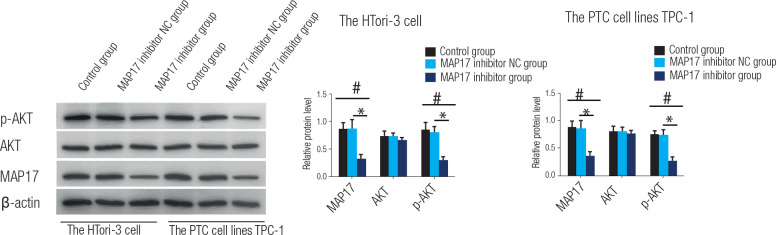
The expression levels of MAP17, AKT, and p-AKT proteins in the HTori-3 cell line and the PTC cell line TPC-1 were determined using Western-blotting. Statistical analysis revealed a significant increase in protein expression levels in the control group compared to the MAP17 inhibitor group (*p < 0.05, #). Additionally, the MAP17 inhibitor NC group showed significantly higher protein expression levels compared to the MAP17 inhibitor group (*p < 0.05).

Western blotting was used to assess the expression levels of MAP17, AKT, and p-AKT proteins in the HTori-3 cell line and the PTC cell line TPC-1, as shown in **Figure 3**. In HTori-3 cells, the findings indicated that the control and MAP17 inhibitor NC groups exhibited significantly higher levels of MAP17 and p-AKT protein in comparison to the MAP17 inhibitor group, with differences being statistically significant (P < 0.05). Conversely, no significant variations in AKT protein levels were observed among the control, MAP17 inhibitor NC, and MAP17 inhibitor groups. Similarly, in TPC-1 cells, both MAP17 and p-AKT protein levels were significantly elevated in the control and MAP17 inhibitor NC groups compared to the MAP17 inhibitor group, with statistically significant differences (P < 0.05). Again, there were no significant differences in AKT protein levels among the control, MAP17 inhibitor NC, and MAP17 inhibitor groups. These results indicate that downregulation of MAP17 protein prevents the occurrence and progression of PTC by controlling the AKT pathway.

### Effect of MAP17, AKT and p-AKT mRNA in PTC

**Figure 4:**
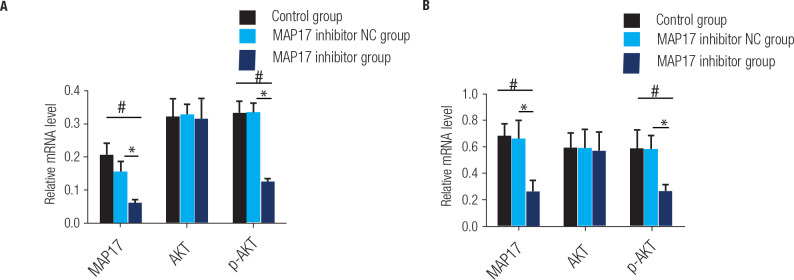
The expression levels of MAP17, AKT, and p-AKT mRNA in the HTori-3 cell line and the PTC cell line TPC-1 were determined using qRT-PCR. Statistical analysis revealed a significant increase in mRNA expression levels in the control group compared to the MAP17 inhibitor group (*p < 0.05, #). Additionally, the MAP17 inhibitor NC group showed significantly higher mRNA expression levels compared to the MAP17 inhibitor group (*p < 0.05).

qRT-PCR was used to assess the expression levels of MAP17, AKT, and p-AKT mRNA in the HTori-3 cell line and the PTC cell line TPC-1, as shown in **Figure 4**. In HTori-3 cells, the results showed that both MAP17 and p-AKT mRNA levels were markedly higher in the control and MAP17 inhibitor NC groups compared to the MAP17 inhibitor group, with the P value indicating statistical significance (P < 0.05). However, there was no significant difference in AKT mRNA levels among the control, MAP17 inhibitor NC, and MAP17 inhibitor groups (shown in **Figure 4A**). Similarly, in TPC-1 cells, both MAP17 and p-AKT mRNA levels were significantly increased in the control and MAP17 inhibitor NC groups compared to the MAP17 inhibitor group, and the P value is Statistical differences (P < 0.05). Again, there were no significant differences in AKT mRNA levels among the control, MAP17 inhibitor NC, and MAP17 inhibitor groups. These results indicate that downregulation of MAP17 mRNA inhibited the occurrence and progression of PTC by controlling the AKT pathway.

## DISCUSSION

PTC is characterized by mutations in key signaling pathways, including the MAPK and PI3K/AKT pathways, both of which are known to play pivotal roles in the regulation of cell growth, survival, and differentiation. MAP17, a small heat shock protein, has been shown to modulate cellular stress responses, and emerging evidence suggests it may also interact with the AKT pathway, contributing to oncogenesis in various cancers, including thyroid carcinoma. Our results align with this view, showing that MAP17 is upregulated in PTC cells and that its interaction with AKT signaling contributes to enhanced cell survival and proliferation. These findings provide new insights into the molecular mechanisms underlying PTC progression, which is crucial for the identification of potential therapeutic targets. However, it is important to consider that while MAP17 and AKT may be involved in tumorigenesis, they do not act in isolation. Other factors, including genetic mutations (*e.g.*, BRAF, RAS), tumor microenvironment, and immune evasion mechanisms, likely interact with these signaling pathways to influence tumor behavior. Therefore, our study represents a piece of the puzzle, but further investigation is necessary to fully understand the complex molecular landscape of PTC and its clinical implications. PTC is a common and usually indolent form of thyroid cancer, but it requires careful evaluation, treatment, and long-term monitoring to ensure optimal outcomes for patients. Currently, various therapeutic methods have been used to treat PTC, but therapeutic effect is limit, and complicated mechanism of PTC led to poor therapeutic effect. More and more studies have indicated that MAP17 and AKT pathway has emerged as a potential player in PTC pathogenesis, their role and mechanism in PTC, yet, were still elusive. Further study should be carried out to clarify the mechanism of PTC. The present trial focuses on investigating the molecular mechanisms of MAP17 in papillary thyroid carcinoma by controlling the AKT signaling pathway. These outcomes suggested that inhibitor of MAP17 can inhibited cell viability in this cell line, while inhibitor of MAP17 can increase the apoptosis level, and the Western blotting and qRT-PCR results showed that MAP17 is involved in PTC by activating AKT signaling pathway. These findings demonstrated the potential targets of MAP17 and AKT in PTC.

MAP17 is a gene that produces a small protein implicated in several cellular functions. This versatile protein is crucial in cancer development and metabolic processes (^16^). Its heightened expression in multiple cancers suggests its viability as a therapeutic target and prognostic marker. Recent studies indicate that MAP17 could be important in the progression of PTC (^9^). Investigating the molecular pathways influenced by MAP17 in PTC and its clinical significance is essential to fully grasp its potential as a therapeutic target and prognostic marker in PTC (^17^,^18^). Yu and cols. (^8^) has investigated the role of MAP17 in PTC, the findings indicated that MAP17 expression was increased in PTC, significantly enhancing the proliferation and movement of PTC cells while suppressing their apoptosis. These results were similar with those reported in our study. Our study results showed that inhibitor of MAP17 can increase the apoptosis level, and upregulation of MAP17 can decrease the apoptosis level. These results indicated that MAP17 is a potential target treatment for PTC.

The AKT signaling pathway, also referred to as the PI3K-AKT pathway, plays an essential role in numerous physiological and pathological processes, such as cell proliferation, growth, survival, metabolic regulation, and cancer progression (^19^,^20^). This pathway is particularly significant in cellular physiology and pathology, especially in the context of cancer development. In PTC cells, activated AKT encourages cell proliferation and prevents apoptosis, facilitating the uncontrollable growth of cancer cells and leading to tumor formation in the thyroid gland (^21^). Research conducted by Hong and cols. (^22^) demonstrated that the AKT pathway is overexpressed and enriched in PTC cells. Our results showed that AKT protein and mRNA levels is clearly increased in PTC cells, and MAP17 participates in PTC process by regulating the AKT pathway. MAP17 influences the regulation of the AKT signaling pathway in papillary thyroid carcinoma, impacting critical cellular processes associated with PTC progression. Gaining a deeper understanding of the interaction between MAP17 and the AKT pathway in PTC may reveal potential therapeutic targets and prognostic markers for this form of thyroid cancer.

We recognize several limitations in our study that should be addressed in future research. First, the sample size used for our experiments was relatively small, which may have limited the statistical power to detect subtle effects. Although we applied rigorous statistical methods (t-test and ANOVA) to evaluate the significance of our findings, small sample sizes can increase the risk of both Type I and Type II errors. Future studies with larger sample sizes and more diverse PTC cell lines or patient-derived models would provide a more robust understanding of the role of MAP17 and AKT in PTC biology. Second, while our study focused on in vitro analyses, the results may not fully translate to in vivo conditions, where the tumor microenvironment plays a significant role in modulating cancer cell behavior. Animal models of PTC would be crucial for validating our findings and understanding the broader biological significance of MAP17 and AKT in the progression and metastasis of PTC. Furthermore, the lack of long-term follow-up in our study means we cannot assess the potential effects of targeting MAP17 and AKT on tumor progression or resistance to therapy.

## Data Availability

the data are free access to available upon request.
